# Open access: What price affordability?

**DOI:** 10.3332/ecancer.2014.ed41

**Published:** 2014-08-14

**Authors:** Richard Poynder

**Affiliations:** Independent journalist and blogger, Cheltenham, UK

Dating from 1665, the scholarly journal has served the research community well for over 300 years. In the past few decades, however, the subscription model it utilises has created a couple of apparently intractable problems—what we will call the *affordability* and *accessibility* problems.

The *affordability* problem has its roots in the dramatic growth in research after World War II, a problem made worse by the constant above-inflation increases in the cost of journal subscriptions—which led to what librarians call the “serials crisis” [1].

The situation was further exacerbated by the fact that learned societies struggled to cope with the growing demand from researchers for publication outlets. Spotting a lucrative market opportunity, for-profit companies (led by Robert Maxwell’s Pergamon Press) began to launch new scholarly journals [2], and to invite learned societies to outsource their journals to them, on the promise that by doing so they would be able to generate more money to subsidise their other activities.

Further aggravating the problem, it turns out that scholarly publishing does not operate like a classic market. For a number of reasons, no effective mechanisms for restraining prices have emerged [3]. In effect, scholarly publishers are as good as able to charge whatever they want for their journals.

The upshot: Today even the wealthiest universities in the world can no longer afford to subscribe to all the journals their faculty need to do their work effectively [4], and the subscription model is now widely viewed as unsustainable so far as scholarly journal publishing is concerned.

While the *accessibility* problem is clearly a consequence of the *affordability* problem (since access would not be an issue if research institutions could afford to subscribe to all the journals they needed), we should view them as separate issues, not least because solving one of the problems will not necessarily solve the other.

It goes without saying that the *affordability* problem is most serious for researchers based in the developing world. Their institutions have never been able to afford more than a handful of journal subscriptions, and the more prices rise the fewer the number of journals they are able to subscribe to, and so the greater the *accessibility* problem becomes for them.

What is the solution to these two problems? Open access advocates believe the answer is for researchers to either continue publishing in subscription journals, but make a copy of their papers freely available to all by depositing them in an OA repository (green OA), or to publish in a gold OA journal by paying an article-processing charge (APC) so that the publisher will make their work freely available for them.

Not only will this solve the *accessibility* problem (since all research will become freely available on the Internet), OA advocates maintain, but it will also solve the *affordability* problem, not least because online publishing is much less costly than print publishing.

But is it possible that while open access may solve the *accessibility* problem, it will fail to solve the *affordability* problem? In embracing OA publishing, for instance, traditional publishers are pricing their APCs not to reflect their true costs, but at a level that will enable them to migrate their journals to an OA environment without suffering any loss of revenue. And OA publishers are increasing their prices in response [5].

Currently it costs around $3,000+ [6] per paper to publish in a subscription journal with an OA option (known as hybrid OA), and it can cost up to $2,885 [7] per paper to publish in an OA journal.

Of course, some OA publishers charge less than this, and some levy no APC at all. However, the growing trend for governments, research funders and universities to create OA funds [8] to encourage researchers to make their work freely available suggests that pay-to-publish gold OA (charged at publishers’ asking price) is set to become the norm. On its current trajectory, therefore, OA looks likely to solve the *accessibility* problem, but not the *affordability* problem.

Once again, it will be researchers in the global South who will be most seriously impacted by this. While OA may enable them to access all the third-party research they want, how will they afford to publish their own research?

Publishers will demur, and point out that developing world researchers can request an APC waiver. However, such waivers are vulnerable to dilution, and perhaps even eventual elimination. It may be that this process has already begun. In 2010, for instance, the non-profit OA publisher PLOS ended its “no questions asked” waiver policy, introducing new eligibility rules. And earlier this year these were further tightened [9].

Meanwhile, applying for an APC waiver from for-profit Elsevier sounds like a decidedly daunting process for impecunious scholars. Its policy [10] reads, “If an author would like their article to be published open access, but cannot afford these fees, then individual waiver requests are considered on a case-by-case basis and may be granted in cases of genuine need”.

Who would relish going cap-in-hand to Elsevier to make a case for “genuine need”? And is it not demeaning to have to ask for charity [11]?

In short, while the promise of OA was that it would resolve both the *accessibility* and *affordability* problems, the clear and present danger today is that it may only address the *accessibility* problem.

And while the developing world may feel the pain most, it will likely only be a matter of time before a new crisis hits scholarly communication in the global North too. How long can it be, therefore, before angry researchers launch a new boycott in the mould of Cost of Knowledge [12], complaining this time not over subscription costs, but the cost of APCs?

But what if funders, governments and research institutions ceased providing money for researchers to pay to publish, and instead insisted that they continue publishing in subscription journals—but always self-archived their papers in OA repositories (green OA)? Would this not mean that publishers would have to compete with repositories in access provision [13]? And would they not as a result lower their prices? And if they did, could we not hope to see both the *accessibility* and *affordability* problems resolved?

Some will respond that in the wake of the pushback [14, 15] against the Finch Report [16], and the subsequent gold OA policy announced in 2013 by Research Councils UK, the trend now is in any case to introduce green OA mandates. But these mandates still sometimes expect researchers to prefer gold OA, and are usually accompanied by APC funds. Moreover, the requirements of a green OA mandate can in any case be met by paying to publish in a gold OA journal. For so long as funders offer to pay their APCs, therefore, most researchers will likely choose that option, if only because it is much easier.

## Conclusion

If greater thought is not given to the implementation of OA it may prove no more sustainable than subscription publishing. In particular, funders and research institutions might want to consider ceasing paying article processing charges for researchers.
